# Review and Update on Some Connections between a Spring-Block SOC Model and Actual Seismicity in the Case of Subduction Zones

**DOI:** 10.3390/e24040435

**Published:** 2022-03-22

**Authors:** Alfredo Salinas-Martínez, Ana María Aguilar-Molina, Jennifer Pérez-Oregon, Fernando Angulo-Brown, Alejandro Muñoz-Diosdado

**Affiliations:** 1 Departamento de Física, ESFM, Instituto Politécnico Nacional, Mexico City 07738, Mexico; asalinasm@ipn.mx (A.S.-M.); anafrom@hotmail.com (A.M.A.-M.); jnnfr.po@gmail.com (J.P.-O.); angulo@esfm.ipn.mx (F.A.-B.); 2 Solid Earth Physics Institute, Department of Physics, National and Kapodistrian University of Athens, Panepistimiopolis, Zografos, 157 84 Athens, Greece; 3 Departamento de Ciencias Básicas, UPIBI, Instituto Politécnico Nacional, Mexico City 07340, Mexico

**Keywords:** self-organized criticality, spring-block, seismicity, Gutenberg-Richter law, asperities, Ruff-Kanamori diagram

## Abstract

The self-organized critical (SOC) spring-block models are accessible and powerful computational tools for the study of seismic subduction. This work aims to highlight some important findings through an integrative approach of several actual seismic properties, reproduced by using the Olami, Feder, and Christensen (OFC) SOC model and some variations of it. A few interesting updates are also included. These results encompass some properties of the power laws present in the model, such as the Gutenberg-Richter (GR) law, the correlation between the parameters *a* and *b* of the linear frequency-magnitude relationship, the stepped plots for cumulative seismicity, and the distribution of the recurrence times of large earthquakes. The spring-block model has been related to other relevant properties of seismic phenomena, such as the fractal distribution of fault sizes, and can be combined with the work of Aki, who established an interesting relationship between the fractal dimension and the *b*-value of the Gutenberg-Richter relationship. Also included is the work incorporating the idea of asperities, which allowed us to incorporate several inhomogeneous models in the spring-block automaton. Finally, the incorporation of a Ruff-Kanamori-type diagram for synthetic seismicity, which is in reasonable accordance with the original Ruff and Kanamori diagram for real seismicity, is discussed.

## 1. Introduction

In recent years, it has been recognized that the earth’s crust is a critically self-organized system, in such a way that seismicity can be studied via the paradigm of a system that exhibits self-organized criticality (SOC) [1]. However, in its beginning seismology was based entirely on an empirical–statistical approach. Some of the first attempts to study the seismic phenomena from theoretical bases were made by Kanamori and Anderson. They designed a dislocation model that considered the accumulated tension and relative sliding between tectonic plates based on relating the energy released, the area of the seismic fault, and the seismic moment, among other physical variables that are involved during the earthquake (eQ) occurrence [2]. Bak, Tang, and Wiesenfeld conceived the idea of SOC systems [3] to describe the global behavior of some complex systems some years later. These authors established the notion of self-organized criticality using a complex system known as the sandpile model [4].

SOC systems allow matter and energy to flow freely between them and their surroundings, and fundamentally, they are made up of many elements with strong short-range interactions and no discernible scale. From a dynamical systems point of view, they can also be understood as a dynamic system in which attractors have a critical point in their dynamics. Sornette and Sornette [5] proposed that SOC may be helpful in understanding earthquake dynamics; Ito and Matsuzaki [6] used the SOC framework to explain several fractal characteristics of earthquakes; and Chen et al. [7] developed a crack propagation model as another SOC application related to earthquakes. All these results support the idea that the phenomenon of seismicity shows SOC behavior.

Olami, Feder, and Christensen (OFC) [8] used the SOC background to create a spring-block system that simulates earthquakes caused by tectonic plate collisions, generalizing the Burridge and Knopoff model [9] into a 2D array. A significant advance was settled as it was possible to reproduce some important relationships of seismicity, such as Gutenberg-Richter’s law, Omori’s law [10,11], and others (see Appendix A), through SOC models that are made up of simple rules which, once they are allowed to evolve, develop complex emergent patterns.

The OFC earthquake model has established itself as a very efficient and popular non-conservative continuous cellular automaton (CA) that models the SOC seismic phenomena. Moreover, it has served as the basis for numerous studies, and many modifications have also been made to obtain more detailed and sophisticated descriptions of seismicity, studying various seismic properties using the spring-block model; for example, understanding the role played by fault heterogeneities has been highly discussed [12,13,14,15,16,17,18]; among the main conclusions, one conclusion is that the fault heterogeneities can play an important role in the frequency–magnitude relations, act as barriers, and intervene in the events clustering.

Fractality is another important studied feature; Main et al. [19] reproduced the fractal distribution of fractures, modeling the positive and negative effects of fluids over failures. Muñoz-Diosdado et al. [20] studied the multifractal structure over a time series, finding monofractality for small conservation levels and multifractality as the conservation level was increased. Gálvez-Coyt et al. [21], using a fractal structure CA, reproduced the Gutenberg-Richter frequency–size distribution law among other seismic properties.

The ability of spring-block models to generate aftershocks as observed in real seismicity (following Omori’s law) has also been addressed by different authors [22,23,24,25,26,27]. Some frameworks involve the modeling of the viscous asthenosphere; for instance, the study presented by Hainzl et al. [22] and the one from Pelletier [23] are both studies capable of reproducing Omori’s law [11]. Many of the most important works on the spring-block models have been the ones able to reproduce the Gutenberg-Richter law, linking seismicity and self-organized critical phenomena, including the works of Bak and Tang [24], Sornette and Sornette [5], Ito and Matsuzaki [6], Sornette et al. [25], Barriere and Turcotte [28], Bak and Chen [29], and Huang et al. [30], among others.

The Gutenberg-Richter law [10] relates the earthquake’s magnitude and the frequency of the events, following the equation:

(1) $$log\dot{N}\left(M\geq M_{0}\right)=a-bM$$

where *M* is the magnitude of the event; $\dot{N}$ is the number of earthquakes per year with a magnitude larger than threshold $M_{0}$; and *a* and *b* are the Gutenberg-Richter parameters.

Spring-block models applied to seismicity analysis are an important tool nowadays; for instance, Hergarten and Jansen [31] used the OFC model in proposing a finite event duration. They found that this changes the general behavior of the OFC model but also concluded that this effect is perceptible only if the rupture propagation is slow. Hence, the usual instantaneous relaxation of the original OFC model is proper.

Varotsos, Sarlis, and Skordas [32] developed the natural time analysis, which allowed them to study the entropy in the OFC model under time reversal, in which they found predictability in this model. Natural time analysis has also been studied in conjunction with nowcasting and synthetic seismic catalogs built through the OFC model in Flores-Marquez et al. [33] and Pérez-Oregon et al. [34], setting good precursory parameters in seismic hazard studies.

Since 1999, our group has worked on the well-known OFC model [8] to emulate the seismicity occurring in subduction zones. In a first stage, this model, and others [35,36], inspired by the pioneering articles by Bak et al. [24,37], obtained synthetic versions of the famous Gutenberg-Richter law. For our part, in 1999 [38], we also obtained this relationship, which statistically characterizes the relation of the frequency of earthquakes with their magnitude. Moreover, through synthetic catalogs, we reproduced the so-called stair graphs, characteristic of the accumulated seismicity over time for several Mexican seismic provinces along the Pacific trench. When these lines move away from the historical slope of accumulated seismicity, they can be interpreted as a period of quiescence (diminished regional seismicity for several years) and probably a precursor to a major earthquake. Quiescence is established for earthquakes with a magnitude greater than or equal to a threshold value [38,39].

In 1999, we found in a second article [39], also using the OFC model, that the distribution of recurrence times of large earthquakes is log-normal, as Nishenko and Buland [40] reported for actual seismicity. Subsequently, in 2012, we published a work [41] in which we proposed some changes to the original OFC model to include the non-homogeneity of the inter-plate interface; this was carried out with sections of different sizes and elastic ratios, as it also was in the homogeneous case.

As we already mentioned, a first step towards establishing that seismicity behaves as an emergent phenomenon from the SOC nature of the Earth’s crust was reproducing the Gutenberg-Richter law using spring-block SOC models. However, it is desirable to try to reproduce other seismicity properties. In 2018, our working group suggested that in the parameters of the GR law, *a* and *b* are positively correlated [42]. For this purpose, we used real seismicity data, as well as data obtained using an OFC-type SOC model. In addition, we presented an analytical demonstration using well-known relationships from the literature.

Recently, we published two works [43,44] in which we were able to reproduce, also using a SOC OFC model, the so-called Ruff-Kanamori diagram [45], in which the magnitude *M_w_* of the maximum characteristic earthquake of a subduction zone is related to the convergence rate *V* between the oceanic and continental plates and the age *T* of the corresponding subducted oceanic lithosphere. We achieved it by adding to the OFC model a way to introduce a variable convergence rate *V* and a method to define a synthetic age through the γ elastic ratio of the model. We believe that this new step provides additional evidence for the SOC nature of seismicity.

In this article, we present a compilation of the main results that our research group has obtained regarding the properties of real seismicity that can be simulated at least qualitatively using the spring-block model and some modifications of it. The properties of real seismicity that can be emulated with this model are discussed in an integrative way in the light of new advances; many of the previous results are updated in this article because we have more computational possibilities to obtain the time series of synthetic earthquakes with millions of events on much larger networks. We wish to highlight that all the calculations that we had carried out in our past publications have been repeated for catalogs of synthetic earthquakes with a much larger number of events, and we report in this article that the results that we obtained have been confirmed: the Gutenberg-Richter law with values of the parameter *b* similar to the real seismicity for values of the *γ* parameter around 0.2; the reproduction of the Ruff-Kanamori diagram; the log-normal distribution of the recurrence times for great magnitude earthquakes; and the ladder-like behavior of the accumulated seismicity. In addition, we have now included a new study of non-homogeneous cases of coupled plates through the variations of elastic ratios along the interface between them.

The article is organized as follows: in Section 2, we briefly present the OFC model and its modifications to obtain other properties of the model that can be observed in real seismicity, such as the Ruff-Kanamori diagram or the inclusion of the asperity concept. In Section 3, we summarize the main results that we have obtained in our leading publications. In Section 4, we discuss them, and finally, we present our conclusions in Section 5.

## 2. Methodology

### 2.1. The Olami, Feder, and Christensen Model

**Figure 1:**
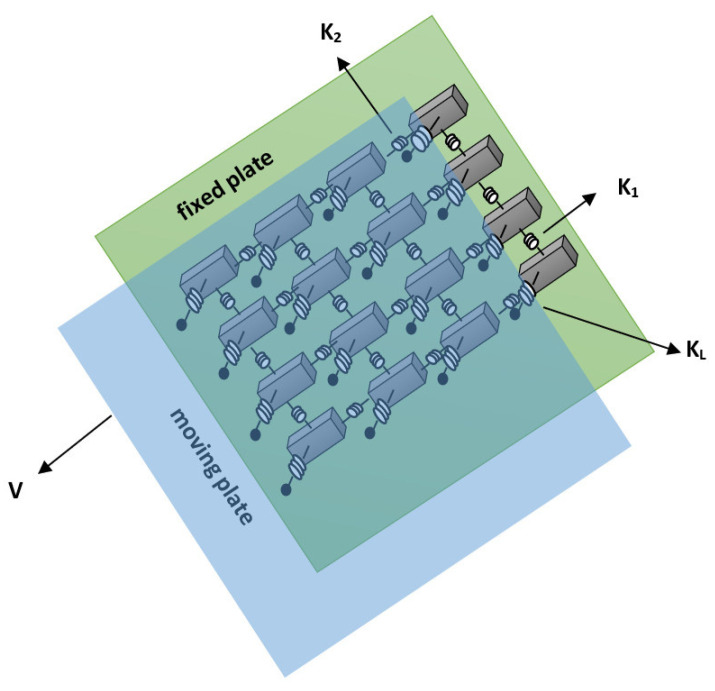
The OFC spring-block model is a two-dimensional dynamic model. Each block is connected to four blocks by springs; below, there is a fixed plate (green), and at the top, it is attached to a movable plate (blue).

The OFC model is a non-conservative continuous model that displays self-organized criticality [8]. It consists of a system of blocks interconnected by Hooke springs. Each block is connected to the four nearest neighbors. In addition, each of these blocks is connected to a moving plate by another set of Hooke springs, and below them, each block is driven with frictional force with the fixed plate, as shown in Figure 1. The relative movement between the two plates results in the block’s displacement; when the force on one of the blocks overcomes a certain threshold *F_TH_* (the maximum static friction), the block slips. The moving block then returns to the zero-force position after transferring its energy to the four nearest neighbors, resulting in further slips that can provoke a chain reaction.

We take the size of a synthetic earthquake as the number *n* of relaxed blocks and use the definition of [43], where magnitude is a function of *n*; for example, $M=log_{r}n$, and *r* is a convenient base of the logarithm.

The cellular automaton model is defined with a matrix of L×L blocks in positions (*I*, *j*), where *i, j* are integers in the interval [1, *L*]. The total force in a block is given by:

(2) $$F_{i,j}=K_{1}\left[2x_{i,j}-x_{i-1,j}-x_{i+1,j}\right]+K_{2}\left[2x_{i,j}-x_{i,j-1}-x_{i,j+1}\right]+K_{L}x_{i,j}$$

where $K_{1}$, $K_{2}$, and $K_{L}$ are the elastic constants.

When the two rigid plates move relative to each other with a relative velocity *V*, the total force in each block increases uniformly with a rate proportional to V until a site reaches the threshold value and the relaxation process begins; then, an earthquake is triggered. The force redistribution after the local slip at position (*i*, *j*) is given by:

(3) $$F_{i\pm 1,j}\to F_{i\pm 1,j}+\delta F_{i\pm 1,j}$$

(4) $$F_{i,j\pm 1}\to F_{i,j\pm 1}+\delta F_{i,j\pm 1}$$

(5) $$F_{i,j}\to 0$$

The force exerted on the nearest neighbors is given by:

(6) $$\delta F_{i\pm 1,j}=\frac{K_{1}}{2K_{1}+2K_{2}+K_{L}}F_{i,j}=\gamma_{1}F_{i,j}$$

(7) $$\delta F_{i,j\pm 1}=\frac{K_{2}}{2K_{1}+2K_{2}+K_{L}}F_{i,j}=\gamma_{2}F_{i,j}$$

where $\gamma_{1}$ and $\gamma_{2}$ are the conservation coefficients or elastic ratios. This *γ*-value determines the amount of energy that is passed to the nearest neighbors, where γ=0.25 would be the maximum percentage that a cell can give to each one of its neighbors, and it would physically mean that there would not be energy loss due to static friction and other inherent mechanisms between the plates. Olami et al. [8] pointed out that when $K_{L}>0$, the distribution of forces is not conservative in the same way as earthquakes occur in nature [8]. There are frictional energy losses in a real earthquake, and not all the energy released contributes to the slip.

The rules for mapping the spring-block model of the cellular automaton are as follows [8]:

(1) Initialize all sites to a random value between *0* and *F_th_*.

(2) If any *F_i,j_* ≥ *F_th_*, redistribute the force on *F_i,j_* to its neighbors according to the rule

(8) $$F_{n,n}\to F_{n,n}+\gamma F_{i,j},F_{i,j}\to 0,$$

where *F_n,n_* are the strains of the four closest neighbors. An earthquake is evolving.

(3) Repeat step 2 until the earthquake has fully evolved.

(4) Locate the block with the highest force *F_max_*. Add *F_th_* − *F_max_*, to all sites (global perturbation), and go back to step 2.

Note that in all synthetic seismicity catalogs it is necessary to discard a non-stationary period until the system reaches SOC behavior. OFC measured the probability distribution of the total number of seismic relaxations that is proportional to the energy released during an earthquake [8].

### 2.2. Asperity Model

In a previous work [41], we proposed a non-homogeneous model taking into consideration the different geological properties in a seismic fault. Our proposal consisted of a grid divided into three parallel regions, where the center has a lower *γ* value than the surrounding regions, as an attempt to introduce in the model the concept of asperity, which is a central concept in real seismicity when considering the subduction between plates.

Nature does not necessarily behave homogeneously, but faults are made up of various components that interact with each other. Therefore, we have also proposed a model where the conservation level is varied, obtaining a non-homogeneous distribution.

In the homogeneous case, where the elastic constants have the same value, $K_{1}=K_{2}=K_{L}\;$, meaning that $\gamma_{1}=\gamma_{2}$, this leads to a value of γ=0.2, which is the best representative value of real-life seismicity [8,41,43]. On the other hand, a model where $\gamma_{1}\neq \;\gamma_{2}$ is considered non-homogeneous. Moreover, it represents the concept of asperity in real life faults. These asperities can be understood as inhomogeneities in the fault, instead of having a single homogenous region. In real seismicity, we can find several regions with different properties and elastic parameters [41].

**Figure 2:**
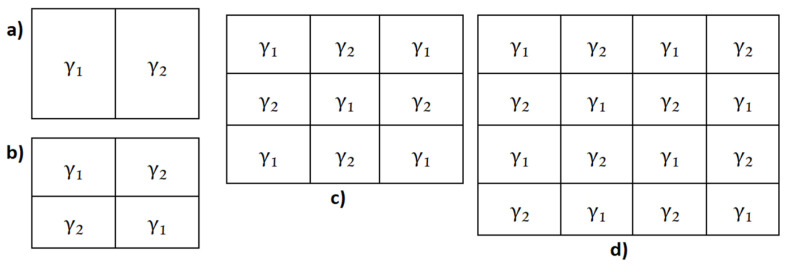
Distribution of the regions in the spring-block matrix: (**a**) 1 × 2 regions, (**b**) 2 × 2 regions, (**c**) 3 × 3 regions, and (**d**) 4 × 4 regions. In all cases $\gamma_{1}=0.2$ and $\gamma_{2}=0.1$.

For the homogeneous, model $\gamma_{1}$ and $\gamma_{2}$ are equal; for the non-homogeneous model, the lattice has been divided into regions with different values of *γ*, as shown in Figure 2. The consequences of this regionalization will be shown later in Section 3.4.

The *γ*-values used for the simulations were $\gamma_{1}=0.2$, which, as we just mentioned, is the best real-life seismicity representative and $\gamma_{2}=0.1$, which represents a region where less intense earthquakes take place due to low energy storage capacity, such as is discussed in references [8,43]. Therefore, an earthquake with an epicenter in a region with $\gamma =\gamma_{1}$ is more likely to propagate to a region with $\gamma =\gamma_{2}$ than vice versa. This $\gamma_{2}$ value was chosen for a comparison between two representative regions with very different level of seismicity.

### 2.3. A Model That Includes the Age and the Rate of Convergence of the Oceanic Plate

Based on the OFC model, we developed a model that included the oceanic plate’s age and convergence rate in a subduction zone [44]. As a result, we not only reproduced the Ruff-Kanamori diagram [45] but also obtained a relation between the elastic ratio *γ* and the age of the tectonic plate *T*.

Taking into consideration that Ruff and Kanamori [45] described different types of coupling between the descending and upper plates, we proposed a case with different *K* values: $K_{1}=K_{2}\neq K_{L}$ and substituting in the γ definition (Equations (6) and (7)), $\gamma =\frac{K}{4K+K_{L}}$, and then setting $x=K_{L}/4K$, which leads to γ≈0.25(1−x). In the plane x vs γ, this Equation represents a straight line with a negative slope, where γ can take values between [0, 0.25], while x is in the interval [0, 1]. In addition, we have some evidence obtained from sandpaper experiments [43,46] where there is a relationship between γ and the age of the weathered surfaces. Considering the interval from 0 to 160 Myr proposed by Ruff and Kanamori [45], we defined $e_{n}$ as the normalized age of the tectonic plates. Assuming that for the maximum value of the tectonic age (160 Myr) it corresponds to the value of 1, it is possible to propose that $x=e_{n}$ to obtain the following equation:

(9) $$\gamma \approx 0.25\left(1-e_{n}\right)$$

We can see that *γ* is related to the age of the tectonic plate; therefore, *γ* is taken as the synthetic age of the tectonic plate. A more detailed explanation of this relation and its physical meaning is presented in Section 3.5.

In a different work [43], for the relative velocity between two plates, we defined a variable Δ*F* (proportional to *V*) as some kind of synthetic counterpart of *V*. In that work, we set this *ΔF* in the interval of [0, 3] and then adjusted the known velocities of the plates between [1, 11.1] cm/yr [44]. Then, we related the lowest interval Δ*F* with the lowest interval of the plate’s velocities and assigned an increase in Δ*F* of 0.25 and an increase of 1 for the velocity, as shown in Table 1.

Finally, to implement the spring-block model, we took the calculated value of *γ* from Equation (9), and Δ*F* was added to the difference between the threshold $F_{Th}$ and the block with the largest force $F_{MAX}$; then, this value was used (only once) as a global perturbation in the first iteration to obtain the biggest event in the simulation [43].

## 3. Results

This section summarizes the main results that our research group has obtained over the years; updates are presented with larger time series and with larger network sizes. The results are presented in an integrative way and discussed considering our new findings.

### 3.1. Power Laws and the Gutenberg-Richter Law

There is a recent consensus [24] that any seismic model must reproduce the Gutenberg-Richter law [10] for the magnitude distribution of earthquakes, but if these models could reproduce at least qualitatively some other properties of seismicity, the models would be more valued. As Fergusson et al. [36] state, if we analyze specific successful models, we can seek knowledge about critical phenomena and arrive at a better theoretical understanding of earthquakes. With this motivation, we have, for several years, examined the OFC model and other variants to find properties that can be associated with actual seismicity [20,21,38,39,41,42,43,44,47,48,49].

**Figure 3:**
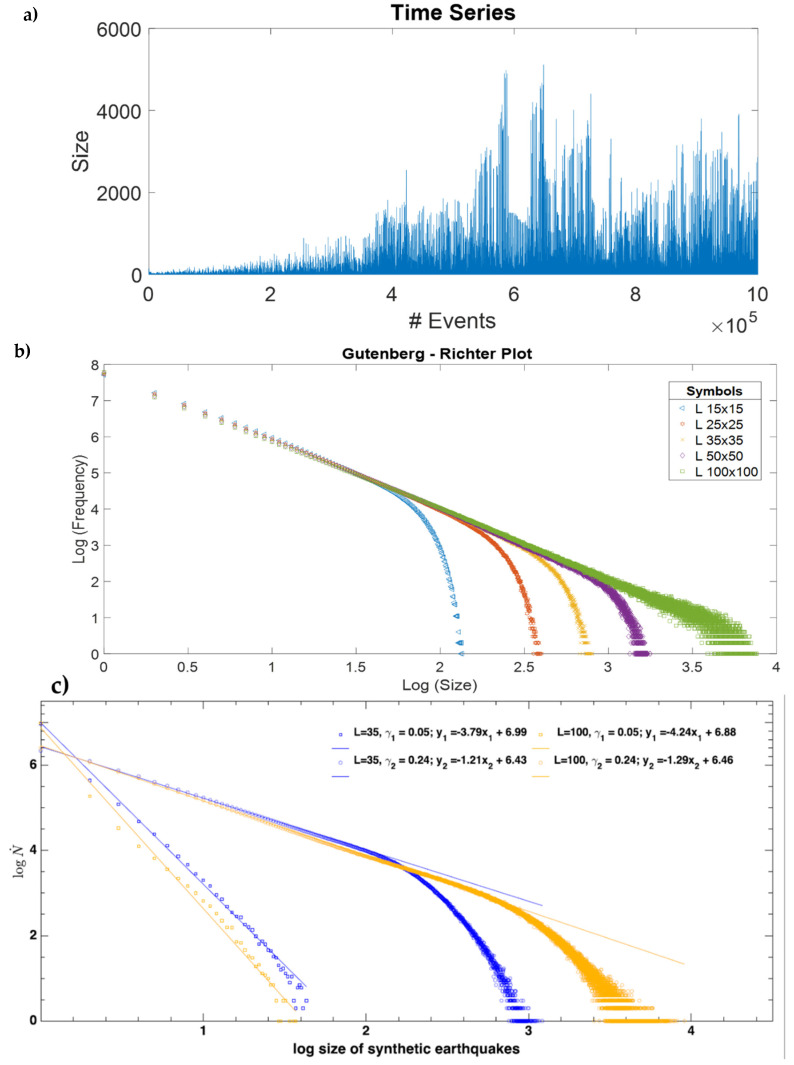
(**a**) Time series for a spring-block simulation (*L* = 100, *γ* = 0.2) of 1 million iterations. We can see a non-stationary behavior during the first 40,000 events prior to reaching a SOC state; the *x*-axis shows the number of events, while the *y*-axis shows the size expressed as the number of blocks disturbed during that event. (**b**) Gutenberg-Richter plot for different lattice values using *γ* = 0.2. From left to right: *L* = 15, 25, 35, and 100. Note the downward curvature due to the finite size effect and the knee shifts to the right as *L* increases. (**c**) Comparison between two extreme *γ*-values, 0.05 and 0.24. *γ* = 0.05 practically does not show a notorious crossover, that is, the finite size effect, even if we change the lattice sizes, in this case *L* = 35 and *L* = 100. For both axes, for simplicity, we used log base 10.

There are many advantages to having a model that reproduces properties of real seismicity, because then synthetic seismicity catalogs could be analyzed without the problems that real seismicity catalogs have. There is no limitation on the number of events that can be simulated in a model, and there would be no overestimation or underestimation problems, and the completeness of the catalog would be guaranteed. For this reason, we proposed that the analysis of synthetic seismicity catalogs, especially those obtained from the spring-block model, can provide us with important information [38,39]. In the articles from our research group, we analyzed only the homogeneous model, that is, when $\gamma_{1}=\gamma_{2}$, and it turns out that synthetic earthquakes satisfy a GR-like law, as can be seen in Figure 3, where the exponent *b* of the Gutenberg-Richter law can be estimated from the slope of the straight line when a least squares fit is performed in the linear region. Note that even though it seems there is only one linear fit for different lattice sizes *L*, a closer look will show small variations in the individual *b* values (slopes) and the *a* values (*y*-intercept). In a later work [42], we studied this variation more deeply, and we found that there is a correlation between these two variables (see Section 3.5). As can be seen for large magnitudes, the graph curves downwards, which is a finite size effect that occurs whenever there is some restriction on the size, as happens in real seismicity where the limitation is the depth of Earth’s crust [49].

Although the GR law (Equation (1)) is universal, the values of the parameters *a* and *b* depend on the subduction region. The constant *a* refers to a regional level of seismicity. Regarding the values of the parameter *b* for real seismicity, there are two regions: the statistics for earthquakes of great magnitude (M>7) give *b*-values between 1.2 and 1.54; while for the small earthquakes, *b*-values approximately between 0.75 and 1.2 are obtained [49]. This may be because a large earthquake can propagate without limitations in the *x* and *y* directions, but in the *z* direction it is limited by the width of the crust [50]. That is, it is the finite size effect. The finite size effect refers to the crossover that can be observed in the Gutenberg-Richter relationship (which in real seismicity occurs around *M* = 7.5) and that denotes a change in the scaling behavior from small and medium earthquakes to large earthquakes. In [50], an explanation for this effect is proposed: in contrast to big events, which are confined by the down-dip breadth of the rupture and therefore do have boundaries in the seismogenic zone, minor events have virtually no bounds inside this zone; so, they do not reach the mentioned crossover (see Figure 3c). This has also been shown in a descriptive scheme presented by these authors (Figure 1 of [50]).

In our synthetic earthquake catalogs, the magnitude of earthquakes follows the GR law (see Figure 3). Interestingly, the OFC model provides *b*-values close to the real values, with values of *γ* around 0.2 [42]. However, the *b*-values obtained for small values of *γ* are much greater than 1.5, and the *b*-values obtained for large values of *γ* are less than 0.75; both cases do not make physical sense because if the value of *b* is very small, then they would produce too many large earthquakes, and if *b* were too large, then the probability of a large earthquake occurring would be very small.

**Figure 4:**
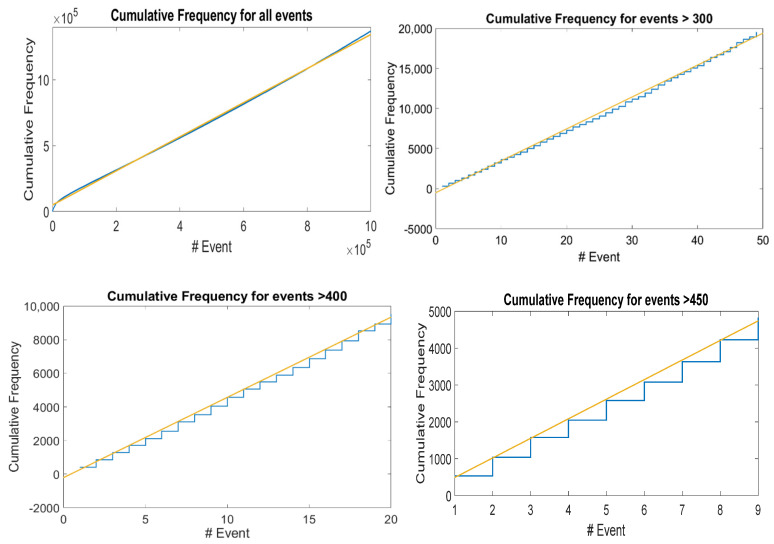
The ladder graphs and the behavior that has been described are also obtained for the accumulated synthetic eQs. (**top**, **left**) Cumulative Frequency of all eQs produced by the spring-block simulation; (**top**, **right**) Cumulative Frequency for eQs bigger than 300 relaxed blocks; (**bottom**, **left**) Cumulative Frequency for eQs bigger than 400 relaxed blocks; (**bottom**, **right**) Cumulative Frequency for eQs bigger than 450 relaxed blocks. In all cases, values of *γ* = 0.2 and *L* = 200 were used. The continuous line represents the historic slope to which they are attracted.

**Figure 5:**
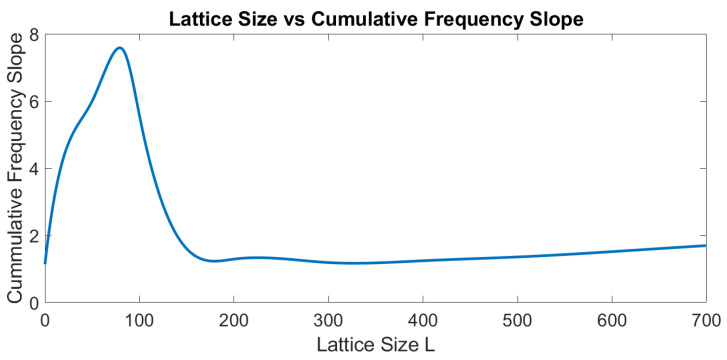
Variation of the slope of the cumulative frequency of earthquakes according to the size of the lattice for *γ* = 0.2. For lattice size < 100, the slope is growing to a maximum value; then, it decreases up to lattice value of 150, and finally, it shows constant growth alongside the lattice’s growth.

In real seismicity, such graphs for cumulative seismicity have been reported by many researchers, such as Habermann and McNally [51,52]. We have also reported such ladder graphs for different seismic regions in the Mexican Pacific Coast [47]; this type of graph has been observed in the subduction zones of the Mexican Pacific of Oaxaca, Guerrero, Michoacán, and Jalisco-Colima. All of them can be characterized by a *b*-value of the Gutenberg-Richter law, and it seems to us that the level of seismicity of such regions can also be characterized with the value of the slope of the long-term straight line. We proposed that this straight-line is a kind of attractor of cumulative seismicity. Therefore, the graph of synthetic seismicity bounded by a straight line is a property of the synthetic cumulative seismicity obtained from the OFC model, and we suggest that it may also be a property of the actual cumulative seismicity. The slope of the line that limits the accumulated seismicity graphs (see Figure 4, top left) depends on both *γ* and *L*; as the values of *γ* and *L* increase, the slope increases, but such a slope cannot be arbitrarily high; for example, in Figure 5, a graph of the slope is shown for different values of *L*. Apparently, the slopes of the lines have a maximum. Therefore, the cumulative synthetic seismicity cannot be arbitrarily large. If the slope of the straight line that limits the graph of the accumulated seismicity were very large, then a small seismic quiescence (horizontal section of a step) could generate an earthquake of great magnitude as the accumulated seismicity tends to return to the mentioned straight line; therefore, there would be too many earthquakes of great magnitude. On the other hand, if the slope of such a straight line were very small, then even if the quiescence were very large, only earthquakes of small magnitude would occur.

### 3.2. Recurrence Times between Earthquakes of Great Magnitude

In real seismicity, when an earthquake of great magnitude occurs in a certain seismic region, the time that elapses until another earthquake of equal or greater magnitude occurs in that same region is called the recurrence time. We call the time that elapses between any two consecutive earthquakes the inter-event time, and the time that elapses between two large earthquakes in the same seismic region is the recurrence time, as with Nishenko and Buland [40].

Brown, Scholz, and Rundle (BSR) [36] proposed a model in which if a large earthquake was preceded by a recurrence time greater than the average recurrence period, then the next large earthquake would be preceded by smaller recurrence time. In the synthetic seismicity catalogs, we obtained the recurrence times in the OFC model by taking a threshold for the magnitude of synthetic earthquakes, in such a way that earthquakes of greater magnitude are preserved.

**Figure 6:**
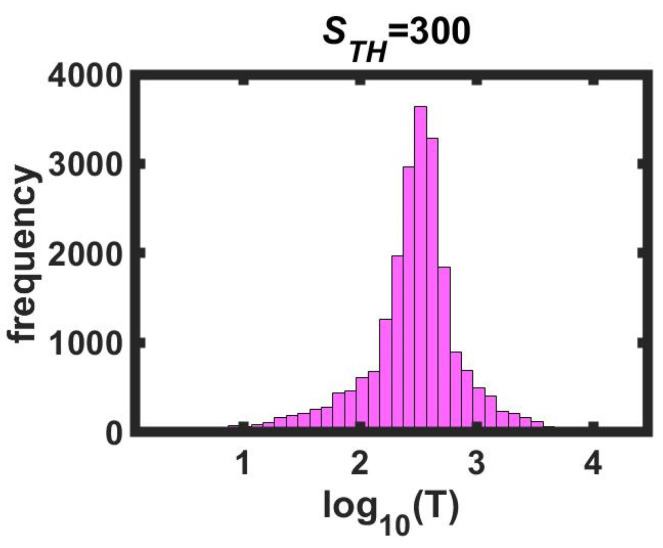
Histogram for the logarithm of recurrence times of synthetic earthquakes of great magnitude, *γ* = 0.2, *L* = 50$,\;N=1\times 10^{7}$ data. In the largest earthquake’s size (*S_Max_*), 1659 blocks were relaxed. In this case, we arbitrarily chose to eliminate all the earthquakes with sizes less than the threshold size *S_TH_* = 300 relaxed blocks. We conserved only 36,230 synthetic earthquakes and calculated the recurrence times. In this case, we have a recurrence time series with 36,230 data points.

Nishenko and Buland [40] analyzed the recurrence times *T_i_* (*i*-th event) normalized by the mean of 50 real large-magnitude earthquakes and found that they had a logarithmic normal distribution. That is, the distribution has the shape of a Gaussian curve and 68 percent of the events were contained within the interval $\bar{x}\pm \sigma$, where $\bar{x}$ is the mean of $\log\left(T_{i}\right)$ and σ is the standard deviation. A synthetic earthquake ends when all the blocks have a stress value below the threshold; the number of computational iterations from when the earthquake begins until it ends is what we call the duration of the synthetic earthquake. The magnitudes and duration times of synthetic earthquakes obtained from the OFC model have a scaling behavior (similar to the GR law), but it was found that the recurrence times do not follow this behavior. For different *γ*-values a different behavior was obtained: sometimes the distribution of recurrence times of large synthetic earthquakes was log-normal, but in other cases, it was not. For small *γ*-values, the distribution is not symmetrical; it is skewed to the left, and for large *γ*-values the distribution is skewed to the right. To summarize, only for *γ*-values around 0.2 did the recurrence times give a logarithmic normal distribution. For an illustration of this behavior, see Figure 6.

### 3.3. The Barriere and Turcotte Model and the Aki Relationship

The Earth’s crust can be described as a hierarchical set of objects of many shapes and sizes that have a fractal distribution [53]. Scale invariance occurs for many geological structures and phenomena, and such self-similar behavior is reflected in the existence of empirical relationships in seismicity [54]. Seismicity has fractal properties concerning magnitude, space, and time [55]. Bak and Tang [24] proposed that both the structure and the dynamics of the crust are the result of a critically self-organized process, and the critical state is characterized by the presence of spatial and temporal power laws. Sornette and Sornette [5] suggested that self-organized criticality helps us to understand earthquakes as a relaxation mechanism that organizes the crust both spatially and temporally.

In 1994, Barriere and Turcotte [28] proposed a model to study synthetic seismicity in which the distribution is fractal; that is, the lattice that represents the seismic fault has fractal geometry. They studied four different lattices with different fractal dimensions. We analyzed the four lattices proposed by Barriere and Turcotte, and we observe that in these lattices the patterns of synthetic seismicity are like the patterns of real seismicity. The synthetic earthquakes observed in this model satisfy the Gutenberg-Richter law; the cumulative seismicity graph has a staircase shape that follows a straight line, and the recurrence times have a log-normal distribution [20,21].

In the Barriere and Turcotte cellular automata model, we change how the magnitude of the synthetic earthquake is defined. In our definition, the magnitude is proportional to the area of the regions that became unstable, in contrast to the Barriere and Turcotte proposal in which the magnitude is the number of particles that leave the fractal cellular automata by the borders [28]; that is, they defined the magnitude like the definition of avalanche size in the sand pile model. Our definition is consistent with reality because the magnitude of an earthquake is proportional to the logarithm of the rupture area. As we reported in reference [21], the relationship proposed by Aki [56], *D* = 2*b*, where *D* is the fractal dimension of the lattice that represents the seismic fault, is approximately fulfilled for the synthetic earthquakes obtained with the de Barriere and Turcotte model.

It was also reported that the duration of synthetic earthquakes follows a power law; so far, there is no confirmation of this fact, but in real earthquakes there are many earthquakes that last a very short time and some that last intermediate times, and there are very few that last a long time, and this is characteristic of the power laws.

### 3.4. Asperities, Non-Homogeneous Spring-Block Model

Byerlee [57] was the first to propose the asperity concept and Scholz and Engelder [58] complimented it; they suggested that the two sides of a fault are held together by surfaces of high resistance, that is, asperities. The stress at the asperities is greater than the average stress across the entire fault plane. There is a random distribution of stresses in various regions of the contact zone. When a strong asperity breaks, a large earthquake occurs, and subsequent breaks occur around the broken asperity. Lay and Kanamori [59] proposed that the asperity distribution is as if the fault zone were subdivided into sub-faults with somewhat independent behavior. We can have multiple regions on the grid that represent the earthquake fault; if we assign a value of *γ* greater than the surrounding area, then that region on the grid can be considered as an asperity. Regions with different sizes and values of *γ* imply different terrain properties. It was reported that in the OFC model large magnitude synthetic earthquakes occur in areas with higher values of *γ*. When a synthetic earthquake occurs, then at a certain point in this area a relaxation occurs (the epicenter), which implies that the next earthquakes will not occur exactly in the same region, but close to it, because there are sites that remained after relaxation very close to the threshold.

If we have a high-stressed asperity near another asperity of the same type and an earthquake occurs in one of them, then it is also possible that an event in one of them could trigger an event in the other [60,61,62,63,64,65,66,67,68].

Several regions can be modeled by putting different values of *γ* in each of the parts into which the grid is divided.

Asperities of various types can be modeled, particularly those described by Lay and Kanamori [59], who observed four types of stress distribution behavior in the fault plane: (a) the regular occurrence of large ruptures of more than 500 km, as in the Nazca fault in Chile; (b) Aleutian-type variation with occasional breaks up to 500 km long and a temporal grouping of large events; (c) the Kurile-type fault in a limited area of 100–300 km in isolated events; and (d) the absence of large earthquakes, as occurs in the Mariana Islands. In the case of the Mexican subduction zones, there is an intermediate case between (b) and (c).

Then, we performed a series of simulations for our own non-homogeneous OFC model. All the simulations were performed with matrix dimensions of 15 × 15, 25 × 25, 35 × 35, 50 × 50, and 100 × 100; these dimensions are expressed as *L* = 15, 25, 35, 50, and 100, respectively, all of them with one million iterations. For all the cases, the Gutenberg-Richter law was obtained.

In Figure 3, we show the behavior of the Gutenberg-Richter law for a homogeneous matrix ranging from *L =* 15 to 100. For small values of *L*, we can observe an abrupt falling from the straight line, forming a “knee”. This knee is the result of the finite size effect inherent in numerical simulations. When increasing the value of *L*, the knee also shows a displacement to the right. As a bigger region is available, the earthquake can evolve to a bigger size before stopping. In comparison, the non-homogeneous model presents the same behavior; it reproduces the Gutenberg-Richter law for earthquakes with very similar values for the slope in each simulation.

**Figure 7:**
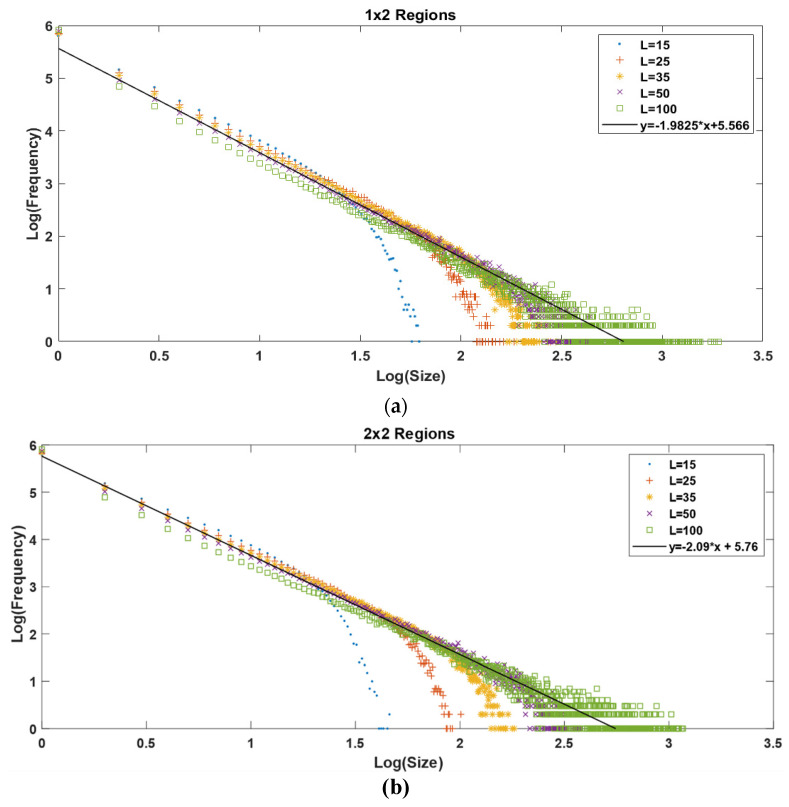
(**a**) Gutenberg-Richter graphic for 1 × 2 regions. (**b**) Gutenberg-Richter graphic for 2 × 2 regions. *L* values correspond to the matrix’s dimensions. For both axes, we used log base 10.

**Figure 8:**
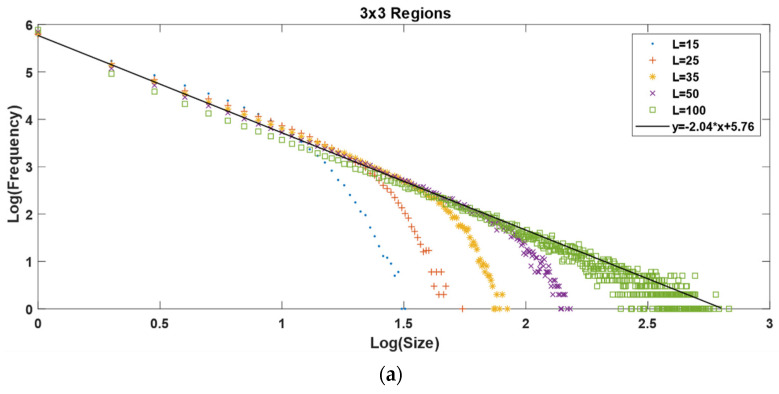

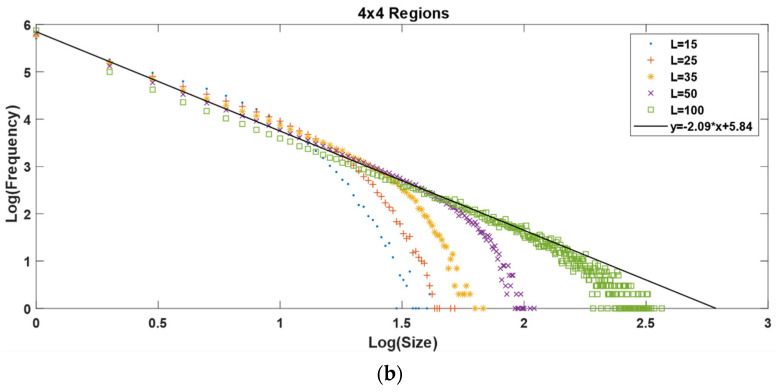
(**a**) Gutenberg-Richter graphic for 3 × 3 regions. (**b**) Gutenberg-Richter graphic for 4 × 4 regions. *L*–values correspond to the matrix’s dimensions. For both axes, we used log base 10.

It is possible to see that when we increase the number of regions, the maximum value of the synthetic earthquake’s size diminishes. The increase in the number of regions goes as follows: one region for the homogeneous matrix and 2, 4, 9, and 16 regions for the 2 × 2, 3 × 3, and 4 × 4 non-homogeneous matrices, respectively. As we can see in Figure 3, the maximum value for the *x* axis, which is also the maximum value for the earthquake’s size in a log-log plot, is ≈3.8; then, when we increase the number of regions to 2, 4, 9 and 16, we obtain a maximum value for the earthquake’s size of 3.3, 3.1, 2.8, and 2.6, respectively, as shown in Figure 7 and Figure 8.

To explain this behavior, we must take into account that even when all the regions have 50% of the matrix’s area with $\gamma_{1}$ and the other 50% with $\gamma_{2}$, their distributions are not the same, giving place to different Gutenberg-Richter graphics. The decrease in the earthquake’s maximum size is due to smaller regions where the energy can be accumulated; this means that, on the one hand, in a 1 × 2 regions matrix with *L* = 100 there is a region of 100 × 50 elements with *γ* = 0.2 where an earthquake can evolve before reaching the other half with *γ* = 0.1; on the other hand, in a 4 × 4 regions matrix with the same *L =* 100, the biggest region for an earthquake to evolve before reaching a lower *γ*-value region is 25 × 25.

Therefore, it is to be expected that the maximum earthquake’s size becomes smaller as the number of regions increases.

For the *a* and *b* values, we can say that *b* does not change significantly when changing the number of regions; it starts at 1.87 for the homogenous case and ends at 2.09 for the 4 × 4 non-homogenous case, which represents a variation of 10%. Comparing only the variations between the non-homogeneous models, *b* goes from 1.98 for the 1 × 2 model to 2.09 for the 4 × 4 model, representing a 5% variation. Regarding the *a*-value, the variations are minimal; it starts at 5.75 for the homogeneous case and ends at 5.84 for the 4 × 4 case, representing a 1.5% variation.

The non-homogeneous spring-block model (Figure 7 and Figure 8) displays the same behavior as its homogenous counterpart (Figure 3); they both reproduce the Gutenberg-Richter law. The finite size effect is present in both models and the displacement of the knee goes from left to right as *L* is increased. The main difference between these two models is the maximum earthquake size they allow.

This non-homogeneous model could be a powerful tool when analyzing seismic zones with asperities or if we had information that allowed us to assume that the energy propagation during an earthquake could be restricted in some directions.

We show that if subdivisions are made in the network that represents the seismic fault, with different sizes and values of the elastic parameters, then the GR and the ladder-shaped graphs of the accumulated seismicity are still obtained, but the process of self-organization lags more than in the homogeneous OFC model; so, the stability of the stepped graphs is also lagged. The slope of the line that delimits the stepped graphs depends on the size *n* of the network, as with the homogeneous case, and always has a maximum global slope. As the results turned out to be like those obtained with real data, we propose that in the future we can have a more suitable model for a seismic fault based on a spring-block model. This is because the concept of asperities can be included in the model through different *γ* distributions.

### 3.5. Correlation between a and b of the Gutenberg-Richter Relation

The following sections, which refer to the correlation between the Gutenberg-Richter parameters *a* and *b* and the study of the convergence rate, age, and largest characteristic magnitude of a seismic subduction zone, are linked by the same common thread, which has led to having, as one of the main results, the reproduction of a synthetic Ruff-Kanamori-type diagram [45] based on the OFC spring-block model. This result is in our consideration particularly relevant for several reasons: in principle, it adds another useful quality to the SOC OFC model, which is able to generate synthetic catalogs that qualitatively reproduce the relationship established in 1980 by Ruff and Kanamori between the maximum characteristic magnitude, age, and convergence rate between the plates. On the other hand, the calculation of these three quantities presents several difficulties; so, having a computational model that allows giving an estimate of any of these values is of great help, considering, of course, that the model itself has its own limitations. Nevertheless, although it is not possible to give precise quantitative results, it gives a point of reference that can be useful for studying these variables. These sections summarize the work published in three articles [42,43,44].

As mentioned, the first of the results found in the use of the spring-block model applied to the seismic phenomenon refers to the positive correlation between variables *a* and *b* of the Gutenberg-Richter frequency–magnitude relationship, a result published in reference [42]. This work started from the positive correlation found from the data reported by Bayrak et al. in 2002 [69]. These authors investigated 27 active seismic zones around the world, reporting, among other information, the *a* and *b* values of the linear fits of the Gutenberg-Richter relationship (*a*, *b*) made for each of the seismic zones under consideration. In a simple evaluation, a=(3.96±0.49)b+(3.62±0.38), and the Pearson correlation of these 27 values was calculated, finding a significant positive correlation (R=0.85, i.e., $R^{2}=0.72$), with a confidence level of 0.05 [42].

**Figure 9:**
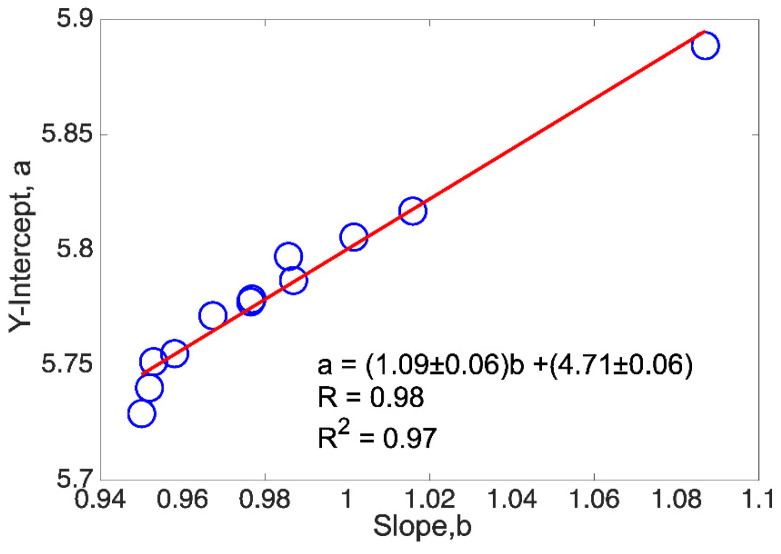
Linear regression in a spring-block case for twelve lattice sizes (*L* = 35, 50, 60, 70, 80, 90, 100, 110, 120, 130, 140, 150) and *γ* = 0.20, as a representative plot. Note that for *L* = 90 and 100 the circles are almost overlapped.

This served as a motivation to verify such a correlation by means of synthetic seismicity catalogs generated from the spring-block cellular automaton. In this way, around 150 synthetic catalogs were manufactured, sweeping a series of cases that allowed a good statistic to be given. High correlation values between *a* and *b* were obtained when the value of the elastic ratio (*γ*) of the spring block was set, and the lattice size was varied (see Figure 9). On the contrary, when gamma varies and the lattice sizes were fixed, several cases were obtained in which the correlation coefficient decreased drastically. However, in general, from the synthetic experiments it was possible to set down that the positive correlation found from real seismic catalogs between *a* and *b* is well-established.

In addition to this, an analytical test was presented in which, starting from some of the best known relationships in seismicity, Gutenberg-Richter’s law, the relation between the magnitude and the seismic frequency [10], Utsu and Seki’s relationship, which relates magnitude and rupture area [70], and Aki’s relation, relating rupture area and fractal dimension [56], the following relation was obtained: a=4b+logC, establishing the positive correlation once again.

Finally, this result (the positive correlation between *a* and *b*) was related to the work of Vargas et al. [46], in which a series of experiments with sliding surfaces were carried out. An aluminum block connected with a fishing string to a motor through a pulley was slid over a frictional surface (experiments with different grades of sandpaper were performed), giving as a result a series of stick–slip event time series reporting the “jumps” that the block made being pulled by the motor overcoming the friction force between it and the rough surface. A very interesting idea arose from this: the spring-block’s gamma friction coefficient varied in a direct way, proportional to the roughness of the sandpaper, so that a small *γ* corresponded to fine-grained sandpaper and a large gamma to coarse-grained sandpaper.

In the experiments in which coarse-grained sandpaper was the initiation point, as it was worn out, this wear meant a decrease in the *γ*-value. This decrease led to the relating of the *γ* coefficient to the age of the plates. So, small *γ* can be related to small friction and large *γ* can be related with large friction. Therefore, small *γ*-values represent older plates while large *γ*-values represent younger plates. This idea of characterizing the age of a tectonic plate from the elastic parameter *γ* was the starting point for the two subsequent works that will be addressed in the next section.

### 3.6. Convergence Rate and Age of Tectonic Plates

This Section summarizes the results we reported in references [43,44]. In those works, we focused on using the spring-block OFC SOC model as a tool to reproduce a Ruff-Kanamori (RK) diagram type for synthetic seismicity. This kind of diagram relates the largest characteristic earthquake magnitude of a subduction zone with the age of the plates and the convergence rate between them. An important consideration is that in the case of synthetic seismicity, the definition of synthetic magnitude was expressed as the logarithm with a convenient base of the size of the synthetic earthquake, which refers to the number of disturbed blocks in the system, and by “convenient” base, we mean one that allows the provision of a feasible comparison between the synthetic magnitude and the real magnitude of an earthquake or event.

Additionally, these works add other contributions. In the first of them [43] we address the analogy that can be established between synthetic seismicity catalogs and experiments with sandpapers. Moreover, we present a brief study within the context of visibility graph analysis (VGA) [71] of the correlation between the *K-M* slope (the variable that relates connectivity and magnitude in the time series of magnitudes) [72] of the synthetic seismic catalogs and the *b*-value of the Gutenberg-Richter relationship. In the second work [44], in addition to proposing an improvement in the plate’s age modeling, we presented an extension of the RK diagram, including some large subduction earthquakes which occurred after 1980.

The qualitative result we obtained in 2018 [42] allowed us to establish the basis of the idea that the elastic ratio *γ* of the spring-block OFC model can emulate an aging effect in the tectonic plates (see Table 2 and Table 3). From this result, the idea of reproducing a synthetic version of the Ruff-Kanamori diagram emerged; nevertheless, a key element was missing: the plate convergence rate or the relative velocity between plates. In this way, we concentrate on proposing a modification to the original spring-block OFC model to be able to involve this physical variable, essential in the dynamics proposed by Ruff and Kanamori, which will be discussed in a little more detail below.

In 1980, Ruff and Kanamori [45] presented a work in which they analyzed several geophysical variables to establish significant correlations between them. They conclude that the age of the subducting oceanic lithosphere (*T*) and the rate of convergence between the oceanic and continental plates (*V*) correlate in a bilinear way with the magnitude of the largest characteristic earthquake (*M_w_*) of a given subduction zone. They also presented the so-called Ruff-Kanamori diagrams that resume this result. Therefore, Ruff and Kanamori were able to establish a bilinear correlation between all these quantities. This correlation was later reproduced in a modified version by Lay and Wallace [73], in which they explicitly give the bilinear relationship (see Equation (10) of Table 4).

In particular, in our work, to model the velocity, a rule was added to the original spring-block automaton proposed by Olami et al. [8]; this was denoted as step 4′ in reference [44], which should be used only if the desire is to emulate an increase in the relative velocity between the plates and which was stated as follows: “Add $\Delta F>F_{th}-F_{max}$ as a global perturbation just for once. Repeat starting from step 2 until the number of events (synthetic earthquakes) is completed.” That is, a surplus delta of energy, greater than the original global disturbance of Olami et al. [8], is induced. Using this proposal, the equivalences between velocity and the deltas of energy or force were established as shown in Table 1.

Then, in order to link the age of the plates with the values of the elastic ratio *γ* and covering the entire age range shown in the zones originally presented by Ruff and Kanamori [45] (ranging from 0 Myr to 160 Myr), a very simple linear relationship was proposed, which constitutes the first approximation to generate a synthetic Ruff-Kanamori- type diagram (see Table 2).

This first approximation to model the age of the plates was used to create the synthetic Ruff-Kanamori diagram reported in [43]. Subsequently, it was in this relationship that an improvement was proposed from a more careful study of the relationship between the elastic ratio *γ* and the elastic constants of the springs that connect them with the mobile upper plate of the physical spring-block model. As we arrived at in Section 2.3, $\gamma =\frac{0.25}{1+x}\approx 0.25\left(1-x\right)$, for a small *x*. This expression of *γ* is our second proposal for modeling a relation between the aging effect through the elastic ratio *γ*. Moreover, we define *x* as e_n_, the normalized age, also defined in Section 2.3.

**Figure 10:**
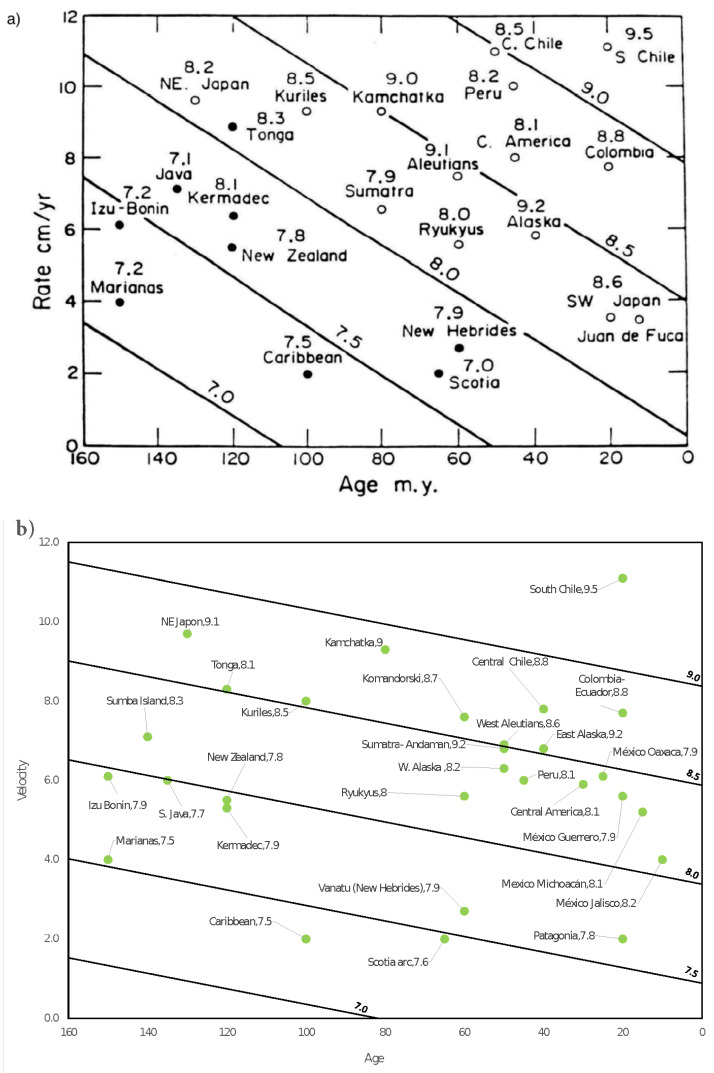

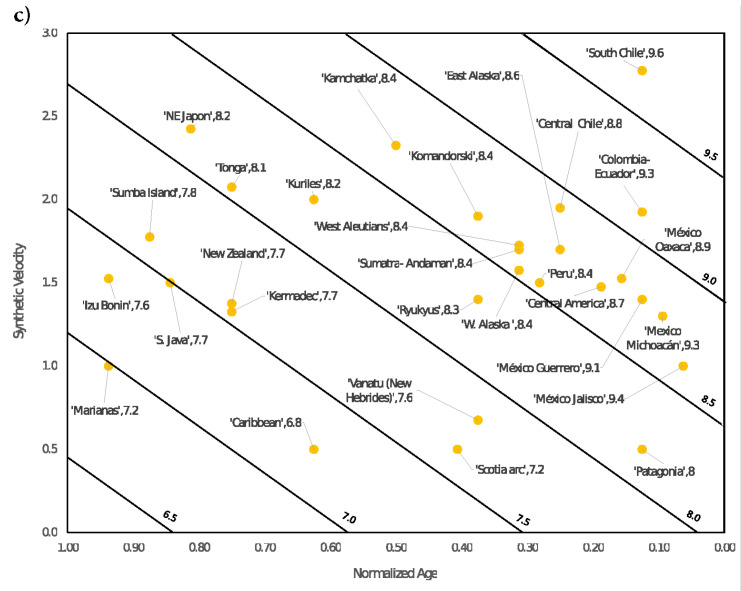
(**a**) Original RK diagram reported in Ruff and Kanamori [45]. (**b**) RK diagram from the extended catalog presented in [44]. (**c**) Synthetic RK diagram from the extended catalog displayed in [44].

**Figure 11:**
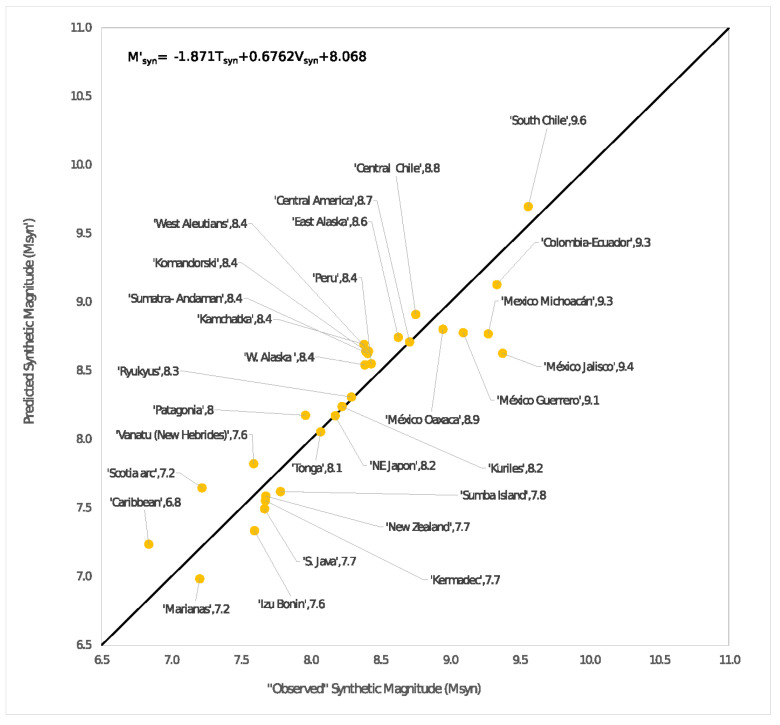
Bilinear fit for the synthetic seismic zones for the extended Ruff-Kanamori using Equation (14).

From this new proposal, the RK diagrams previously presented were recalculated, obtaining a closer qualitative agreement between the new synthetic RK diagram and the original presented by Ruff and Kanamori [45]. As it was mentioned, the RK diagram of real seismicity was also extended considering seven new regions: Komandorski, Patagonia, Sumba Island, México-Jalisco, México-Michoacán, México-Guerrero, and México-Oaxaca and Alaska, which was divided into East Alaska and West Alaska. From this new set of subduction zones, the RK diagrams were re-manufactured for the real seismicity and for the synthetic case (see Figure 10); all the bilinear fits were summarized in Table 4, and Equation (14) is shown in Figure 11. All this, in addition to having the intention of an update of the diagram proposed by Ruff and Kanamori, also helped to reinforce the reproduction of an RK diagram type through a spring-block modified model.

It is interesting to note that in reference [74] Kanamori states that the bilinear relationship given in Equation (10) of Table 4 was obtained empirically without using any physical model. On the other hand, Scholz [75]) says that on balance it seems that seismic coupling is positively correlated with *V* and *T* only when they are taken together. That said, the reproduction of the RK diagram published in our works [43,44] could be the first time that it has been achieved using a physical model (modified OFC model).

## 4. Discussion

To summarize, for values of *γ* around 0.2, the OFC model provides values in good concordance with the *b*-parameter of the Gutenberg-Richter law, which coincides with the values obtained from the real seismicity, and a log-normal distribution of the recurrence times is obtained for the largest earthquakes.

Furthermore, this model has shown other properties related to real seismicity, such as ladder-shaped graphs for cumulative seismicity, which allow us to study patterns of seismic quiescence as possible precursors of large EQs. We find that the synthetic cumulative seismicity in the long-term situation is limited by a straight line whose slope depends on the size of the system, and this slope cannot be arbitrarily large.

It is interesting to note that these properties are observed both in the homogeneous OFC model (a single *γ*-value in the entire network representing the seismic fault) and in the non-homogeneous model (the network representing the fault is subdivided into different regions with different *γ*-values). The homogeneous model already has certain inhomogeneities as the boundaries are open, as reported in one of our previous works [41]. The blocks that are at the edge of the network have different behavior from the rest of the blocks. We showed in [41] that this fact produces a qualitatively similar behavior to that reported by Corbi et al. [76] for subduction faults. Most of the global seismic energy is released by sliding between the subducting and the upper plates. Corbi et al. [76] reported that the interplate contact is seismogenic only in a specific depth interval, globally comprised between 11 ± 4 and 51 ± 9 km. The accumulated seismic moment is not homogeneously distributed along the seismogenic zone; it shows an approximately Gaussian distribution with a peak around 20–30 km in depth. We mentioned that this is physically reasonable because the interplate is confined between the surface and the mantle; so, large earthquakes have no limits on rupture length, but their down-dip width is limited by the thickness of the region capable of generating earthquakes [50]. We showed that the OFC spring-block homogeneous model qualitatively reproduces this behavior [41].

Moreover, as an application of the inhomogeneous OFC model, we reported the qualitative reproduction of a seismicity pattern observed in the Guerrero gap region in Mexico [41]. Suárez et al. [77] found that in the Guerrero gap the seismicity is concentrated in two parallel regions, and between them, there is another region with almost no seismicity. We have reproduced this pattern qualitatively and suggested the presence of two great asperities that surround the quiet zone.

Seismology has lacked a theoretical basis to explain empirical power laws, such as Gutenberg-Richter’s law, Omori’s law, and Utsu’s relationship. It was until the emergence of the SOC concept in 1987 [3] that the empirical power laws were derived from many models, the OFC model being one of the most important SOC models.

Regarding the latest results of the application of the spring-block OFC model to the seismic phenomena carried out in our group, we can highlight the following: in reference [42], it was possible to establish that there is a correlation between the *a* and *b* parameters of the Gutenberg-Richter frequency–magnitude relationship, where these parameters are the variables of the linear fit of this relationship. This result was concluded from four different frameworks: real seismicity (from seismic catalogs covering 27 seismic zones around the world [69]), synthetic seismicity (coming from the spring-block model), laboratory experiments with a table covered with a sandpaper track [47], and an analytical demonstration [42]. In all these cases, the positive correlation between *a* and *b* was verified.

In the case of synthetic seismicity, the highest values of the Pearson coefficient *R* and the coefficient of determination ($R^{2}$) were obtained for the interval *γ* = [0.18, 0.21], in which, remarkably, the value *γ* = 0.20 is included. This *γ*-value has been pointed out by different authors [8,38,50] as the elastic ratio value that better reproduces the statistical features of real seismicity. However, it is also relevant to mention that in this study we also found intervals of values in which the correlations were not significant; this occurred in the cases in which the lattice size *L* was fixed, and the interval of the elastic ratio was varied. According to the study carried out, it seems that the scenarios in which *γ* is fixed and *L* is varied (which report higher values of *R* and $R^{2}$) are the ones that better represent what is exposed in the real seismic phenomenon, implying a diversity of sizes in the seismic provinces.

Regarding the stick–slip experiment with sandpapers, the qualitative behavior obtained could be reproduced by extreme values of the elastic ratio in the OFC spring-block model. This result can be linked to the explanation by means of the so-called finite size effect presented in [50], and importantly, the value of the elastic ratio *γ* can be used to emulate the aging effect in plate tectonics.

Concerning our last two works [43,44], these are intrinsically related, as we explained in Section 3.5. The most substantial contributions in those are: in principle, the proposal of a modification to the original spring-block OFC model that allows simulating the velocity between plates, which later made it possible to obtain synthetic versions of the Ruff-Kanamori diagram that links the maximum characteristic earthquake of a subduction zone with the age and convergence rate of the plates of the zone under consideration. Finally, we have presented an extension of the original diagram of Ruff and Kanamori [45] of real seismicity, in which seven new seismic regions were added to the study. The synthetic seismicity diagrams produced were able to describe the qualitative behavior of the original RK diagram.

The extreme cases of these diagrams are the clearest to visualize in this regard. According to the original RK diagram, Mariana’s seismic zone which is an old plate (150 Myr) and, in addition to its low convergence rate (4.0 cm $y^{-1}$), allows a combination that results in a maximum characteristic earthquake with a magnitude of 7.2. In counterpart, the South Chile region is a very young plate (20 Myr) that has a high convergence rate between plates (11.1 cm $y^{-1}$), resulting in a characteristic maximum earthquake magnitude of 9.5.

This phenomenology is analogously reproduced with the synthetic diagrams in the improved version [44] because, as explained in Section 3.5, in [43] a first approximation was exposed in the correspondence between the values of the plate age and its corresponding elastic ratio *γ*, according to which Marianas presents a characteristic maximum synthetic earthquake of 7.2 (*γ* = 0.016 (*en* = 0.94), Δ*F* = 1.0), while Southern Chile obeys *M_max_ =* 9.6 (*γ* = 0.219 (*en* = 0.13), Δ*F* = 2.775). Another relevant element to mention is that the aging effect observed in the sandpaper experiments can be expressed in terms of the anti-correlation presented by the parameter *b*-GR and the elastic ratio *γ*.

Lastly, we want to emphasize that the qualitative behavior of the RK diagrams, which relates the magnitude *M_w_* with the convergence rate *V* and the age of the tectonic plate *T*, was established as a bilinear form, in which, for both the real and the synthetic cases, an anti-correlation between *M_w_* and *T* can be set (numerically expressed with *A* < 0) and, in counterpart, with a positive correlation between *M_w_* and *V* (numerically expressed with *B* > 0, where *A* and *B* are the regression polynomial coefficients).

In consideration of all these results together, we can conclude that it could be suitable to tell whether the seismicity in the subduction zones follows a true SOC pattern if we start from a large enough set of maximum characteristic earthquakes and combine it with trustworthy data of the age and convergence velocity rate of tectonic plates.

## 5. Conclusions

As is well known, macroscopic systems in thermodynamic equilibrium give rise to emergent properties, some as familiar as the pressure and temperature of a gas. On the other hand, in nature complex systems abound that are out of thermodynamic equilibrium, being able to exchange matter and energy permanently with their surroundings. These systems cannot reach true states of stable equilibrium but only metastable states; however, these systems also generate emergent properties. The seismicity that occurs mainly in the earth’s crust can be considered as an emergent property of the complex system that is the earth’s crust that exchanges matter and energy mainly with the earth’s mantle.

Since Bak et al. [24] coined the concept of critically self-organized systems, one of the most studied phenomena in the context of this methodology is seismicity. In this article, we have gathered and updated our results in an integrative approach to help increase the evidence that the dynamics of seismicity is most likely an SOC-type dynamics by using better computational tools. Thus, with much larger synthetic catalogs than we had in our previous publications and much larger sizes of the network representing seismic faults, we were able to confirm again what we had already reported.

Among our most relevant results is the reproduction of Ruff-Kanamori diagrams using the OFC spring-block model, not to mention obtaining the distribution of recurrence times for large earthquakes that are log-normal, as reported by Nishenko and Buland [40] for real earthquakes. In this work, we also show how, using the OFC model, the concept of asperities can be incorporated into the study of seismic dynamics. This modified OFC model allows the modeling of the force distribution alongside different elastic coefficients within the subduction regions or zones where the energy propagation could be restricted in some directions.

## Figures and Tables

**Table 1:** The left column represents the velocity range reported in the original RK diagram of Ruff and Kanamori (1980), while the right column represents the corresponding Δ*F*-value [44].

Velocity (cm/Year)	Δ*F*-Values
1	0.25
2	0.5
3	0.75
4	1
5	1.25
6	1.5
7	1.75
8	2
9	2.25
10	2.5
11	2.75
12	3

**Table 2:** The left column represents the age range reported in the original RK diagram of Ruff and Kanamori (1980), while the right column represents the corresponding *γ*-value.

Age (Myr)	*γ*-Values
20	0.20
30	0.19
40	0.18
50	0.17
60	0.16
70	0.15
80	0.14
90	0.13
100	0.12
110	0.11
120	0.10
130	0.09
140	0.08
150	0.07

**Table 3:** In the first column, the lithospheric age range is given as it is reported in the original RK diagram of Ruff and Kanamori [45]. In the second column, the *γ*-values of the OFC model are shown, which emulate the aging effect, and in the third column, the normalized ages are given.

Age *T* (Myr)	*T_syn_* (*γ*-Values, Dimensionless)	*T* Normalized (*e_n_*, Dimensionless)
20	0.219	0.125
30	0.203	0.188
40	0.188	0.250
50	0.172	0.313
60	0.156	0.375
70	0.141	0.438
80	0.125	0.500
90	0.109	0.563
100	0.094	0.625
110	0.078	0.688
120	0.063	0.750
130	0.047	0.813
140	0.031	0.875
150	0.016	0.938

**Table 4:** Bilinear fits for the different cases studied. In all cases, *M* represents the largest characteristic earthquake. *M’_w_* refers to the magnitude calculated by Lay and Wallace [73] from real seismicity data, where *T* (Myr) is the age of the corresponding subducted oceanic lithosphere, and *V* (cm/y) is the convergence rate between the oceanic and continental plates. *M’* is the magnitude calculated by us, extending Ruff –Kanamori’s work [45] by considering more seismic zones around the world. *M’_syn_* is the synthetic magnitude, i.e., the magnitude calculated from synthetic catalogs performed through the spring-block model. In the same way, *T_syn_* is the synthetic age (the *γ*-values given in Table 3), and *V_syn_* is the synthetic convergence rate (the Δ*F*-values given in Table 1). Note the similarity between Equations (12) and (14).

Linear Multivariate Fit	Equation	
Original Ruff-Kanamori (Performed by Lay and Wallace [73])	$M_{w}^{\prime }=-0.00983T+0.143V+8.01$	(10)
Synthetic Ruff-Kanamori type (First proposal for aging effect)	$M_{syn}^{\prime }=-4.32623T_{syn}+0.957V_{syn}+6.78$	(11)
Synthetic Ruff-Kanamori type (Second proposal for aging effect)	$M_{syn}^{\prime }=-1.3662T_{syn}+0.653V_{syn}+7.73$	(12)
Extended Ruff-Kanamori proposal (Real seismicity)	$M^{\prime }=-0.003909T+0.2003V+7.32$	(13)
Synthetic case for the extended Ruff-Kanamori	$M_{syn}^{\prime }=-1.871T_{syn}+0.6762V_{syn}+8.068$	(14)

## Data Availability

Both real and synthetic seismicity data can be provided by writing to the corresponding author.

## Appendix A. Empirical Laws of Seismology

*Gutenberg-Richter’s Law*

The Gutenberg-Richter law [10] relates the earthquake’s magnitude and the frequency of the events, following the equation:

(A1) $$log\dot{N}\left(M\geq M_{0}\right)=a-bM$$

where *M* is the magnitude of the event; $\dot{N}$ is the number of earthquakes per year with magnitude larger than threshold $M_{0}$; and *a* and *b* are the Gutenberg-Richter parameters.

*Omori’s law*

Omori’s law [11] states that on average the aftershock frequency decreases hyperbolically with time after a strong earthquake, following the equation:

(A2) $$n\left(t\right)=\frac{k}{\left(\rho +t\right)}$$

where *n(t)* is the aftershock frequency; *k* is the rate of aftershock decay; *t* is the time; and ρ is a constant.

*Aki’s Relation*

Aki [55] proposed that there exists a relationship between the fractal dimension of the spatial distribution of earthquakes and the *b*-value of Gutenberg-Richter, given by the following equation:

(A3) $$D=\frac{3b}{c}$$

where *D* is the fractal dimension, and *c* is the slope of the linear relation of the logarithm of the seismic moment vs. the earthquake magnitude, which is approximately 1.5, leading to:

(A4) D=2b

*Utsu’s law*

Utsu proposed a relation between the earthquake’s magnitude and the area in which the main event’s aftershocks may occur [78], according to the equation:

(A5) Log A=1.02 M−4.01

where A is the aftershock area in km^2^, and M is the earthquake’s magnitude.
